# Findlater jet induced summer monsoon memory in the Arabian Sea

**DOI:** 10.1038/s41598-022-17025-1

**Published:** 2022-07-29

**Authors:** Vikas Kumar Kushwaha, S. Prasanna Kumar, F Feba, Karumuri Ashok

**Affiliations:** 1 Centre for Earth, Ocean and Atmospheric Science, University of Hyderabad, Hyderabad, India; 2 CSIR-National Institute of Oceanography, Dona Paula, Goa 403002 India

**Keywords:** Ocean sciences, Climate sciences, Ocean sciences

## Abstract

A cross-equatorial low-level wind, known as Findlater Jet (FJ), modulates the thermocline in the Arabian Sea (AS) during summer monsoon (June to September). By analysing ocean and atmospheric data, we show that the FJ signal gets ‘trapped’ in the AS in the form of upper ocean heat content till the following winter months (December to February). This memory is the consequence of the combined effect of FJ-induced wind stress curl and the annual downwelling Rossby waves in the AS. During the summer monsoon months, the strong low-level westerly winds cause a negative wind stress curl in the south of the FJ axis over the central AS, resulting in a deep thermocline and high magnitude of heat being trapped. In winter monsoon months, though the wind stress curl is positive over large parts of the AS and could potentially shoal the thermocline and reduce the upper ocean heat content in the central AS, this does not happen due to two reasons. Firstly, winds are weaker, and spread over a larger area over the AS making the magnitude of the wind stress curl low. Secondly, westward propagating downwelling Rossby wave radiated from the eastern AS deepens the thermocline and prevents ventilation of the trapped heat. During the following spring, the collapse of the Rossby waves leads to the shoaling and mixing of underlying waters with surface waters thereby resurfacing of the trapped heat. The resurfacing of the trapped heat makes the AS a memory bank of the FJ induced signal.

## Introduction

The Indian Summer Monsoon (ISM), also known as southwest monsoon, is a prominent ocean-atmospheric phenomenon characterized by organized south-westerly winds and enhanced rainfall that becomes active during June to September. It affects the livelihood of the people of the Indian subcontinent and also impacts the dynamics and biogeochemistry of the surrounding water bodies, the Arabian Sea (AS) and the Bay of Bengal (BoB), making their regional oceanography contrastingly different^1^. The ISM accounts for 70% of annual precipitation over India and 60% of agriculture sector jobs^2,3^. Though the interannual variability in the ISM rainfall is small with a standard deviation of about 10%, it can severely affect the economy of this region which is largely based on rain-fed agriculture and industry based on agriculture. Understanding the effects of environmental change on ISM rainfall and its spatial patterns present a key research challenge^4^ with huge ramifications on the water resources and the management policies. Thus, a lead prediction of the ISM turns out to be exceptionally critical and important.

The AS has a strong seasonality wherein the atmospheric as well as the oceanic circulation switches directions semi-annually under the influence of seasonally reversing monsoon winds. During ISM the cross-equatorial atmospheric flow from the East African coast towards India, the south-westerly winds, develops by the end of May, intensifies into a low-level jet during July, and collapses by end of September^5,6^. This low-level atmospheric jet, known as Findlater Jet^7^ (FJ), attains a speed as high as 100 knots near the East African coast^8,9^. The axis of the FJ is represented by the region of maximum wind speed at a height of 850 millibars (hPa) and extends from the Horn of Africa to the coast of Gujarat in India. The FJ plays an important role in the spatio-temporal variability of the ISM rainfall. For example, Webster et al.^2^ and Pushpanjali et al.^10^ found that the SM rainfall is positively correlated with the strength of FJ. In general, strong FJs are associated with more active spells of ISM rainfall, while weak FJs are associated with breaks in the rainfall. The characteristics of the FJ and its variability ranging from diurnal^11–13^, intraseasonal^14,15^, and to interannual^10,16,17^ time scales has been well-researched. There are also studies on the long-term trends of FJ^18,19^.

It is known that FJ has some influence on the upper ocean through wind stress. The cyclonic wind stress north of the axis of the FJ induces Ekman suction and open-ocean upwelling, while the anticyclone wind stress south of the axis leads to Ekman pumping and open ocean downwelling^20,21^. This results in the modulation of mixed layer depth on either side of the axis of the FJ^22–24^. During the winter monsoon months (December to February; DJF), though the north-easterly winds are weak the convective cooling and associated mixing deepens the mixed layer in the northern AS^23,25^. The other dominant factor influencing the upper ocean including the mixed layer in the AS is the annual Rossby waves^24,26,27^. They are the dominating patterns of sea surface height (SSH) variability, especially all through the inter-monsoon period. The westward propagation of first—and second mode annual Rossby waves explains 87% of the seasonal, mid basin hydrographic variance below 100 m, along 8°N^26^.

Though there had been various studies focused on understanding the dynamics of circulation in the north Indian Ocean during different seasons^28,29^ there are only a few studies that have focused on the impact of FJ on the modulation of the upper waters in the AS during the ISM months^20,21^. However, no studies have examined the impact of the FJ on the subsurface heat content in the AS beyond the ISM months and its feedback on the ISM rainfall. Therefore, the main objective of the current study is to explore the response of the subsurface AS to the FJ and examine potential processes in regulating ISM rainfall through oceanic heat content changes.

## Results and discussion

**Figure 1:**
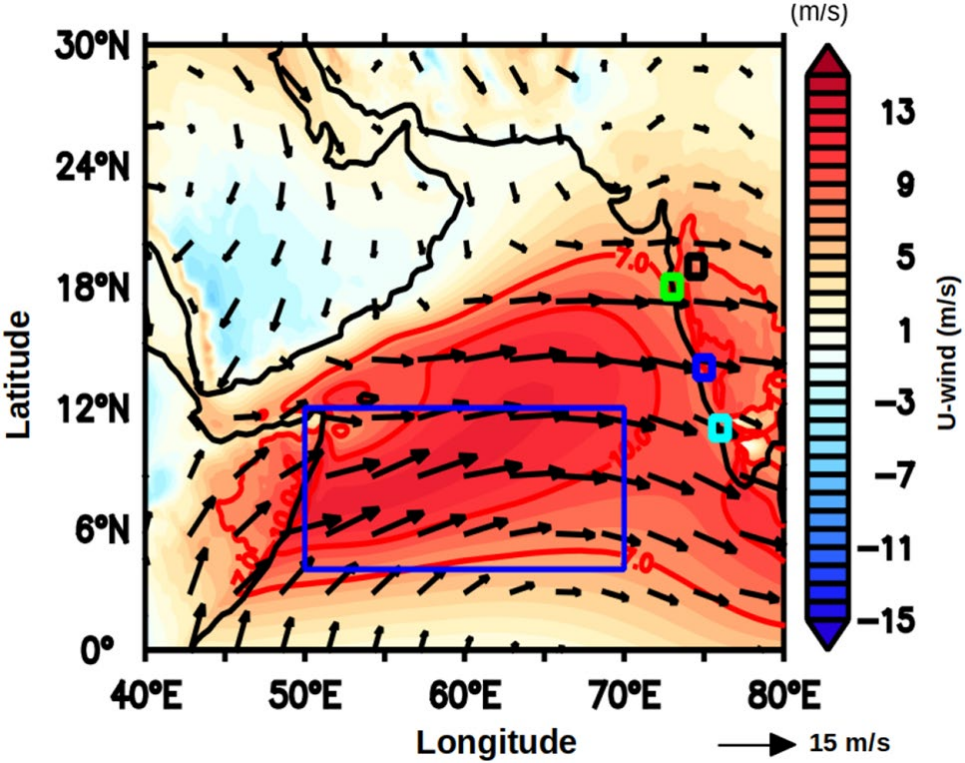
Climatological mean wind vector overlaid on zonal wind (u-wind, m/s) during June to September (JJAS) at 850 hPa in the Arabian Sea. The blue box (50–70°E and 4–12°N) represents the region within which parameters were averaged. Red solid lines indicate the zonal wind speed contours of 7 m/s and 10 m/s. The different colour small squares represent the location of sub-divisonal stations, Madhya Maharashtra (black), Konkan (green), Coastal Karnataka (blue), and Kerala (cyan).

**Figure 2:**
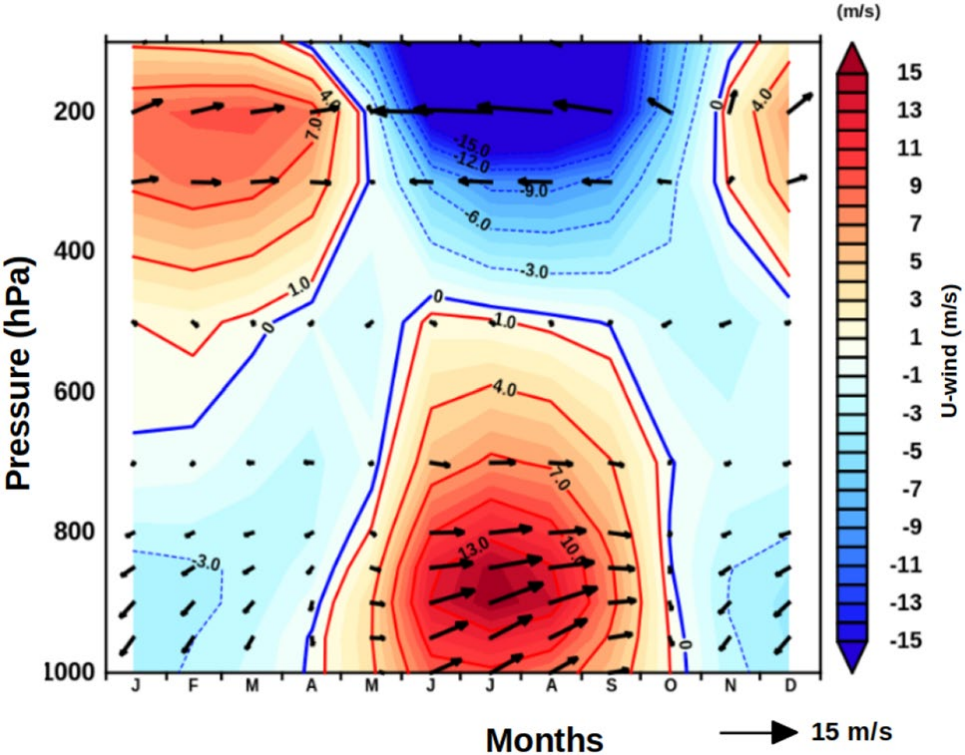
Vertical structure of monthly mean climatology of zonal wind (u-wind, m/s) averaged over the longitude 60°E to 70°E and latitude 6°N to 20°N overlaid with wind vectors.

In order to understand the role of FJ in regulating the ISM rainfall, the climatological mean wind at 10 m above the sea surface for the study period was examined during JJAS (Fig. 1), which shows the signature of the FJ as a core of strong south-westerly winds. The presence of the FJ can be identified by the climatological south-westerly winds seen over the central AS with a zonal wind speed in excess of 10 m/s. Figure 2 shows the temporal and vertical structure of the wind vectors averaged over the box (Fig. 1) located in the core region of the FJ in the central AS. During the ISM, the maximum intensity of the FJ is seen at 850 hPa. These winds are mostly westerly up to the height of 600 hPa, and the winds reverse to easterlies at greater heights with an increase in their magnitude. Just after the JJAS, the ISM winds collapse, and the north-easterly winds start to prevail prominently between 1000 and 850 hPa indicating the winter monsoon conditions. Notice that the upper-level easterly winds between 200 to 300 hPa of the ISM also reverse to westerly during the winter monsoon.

**Figure 3:**
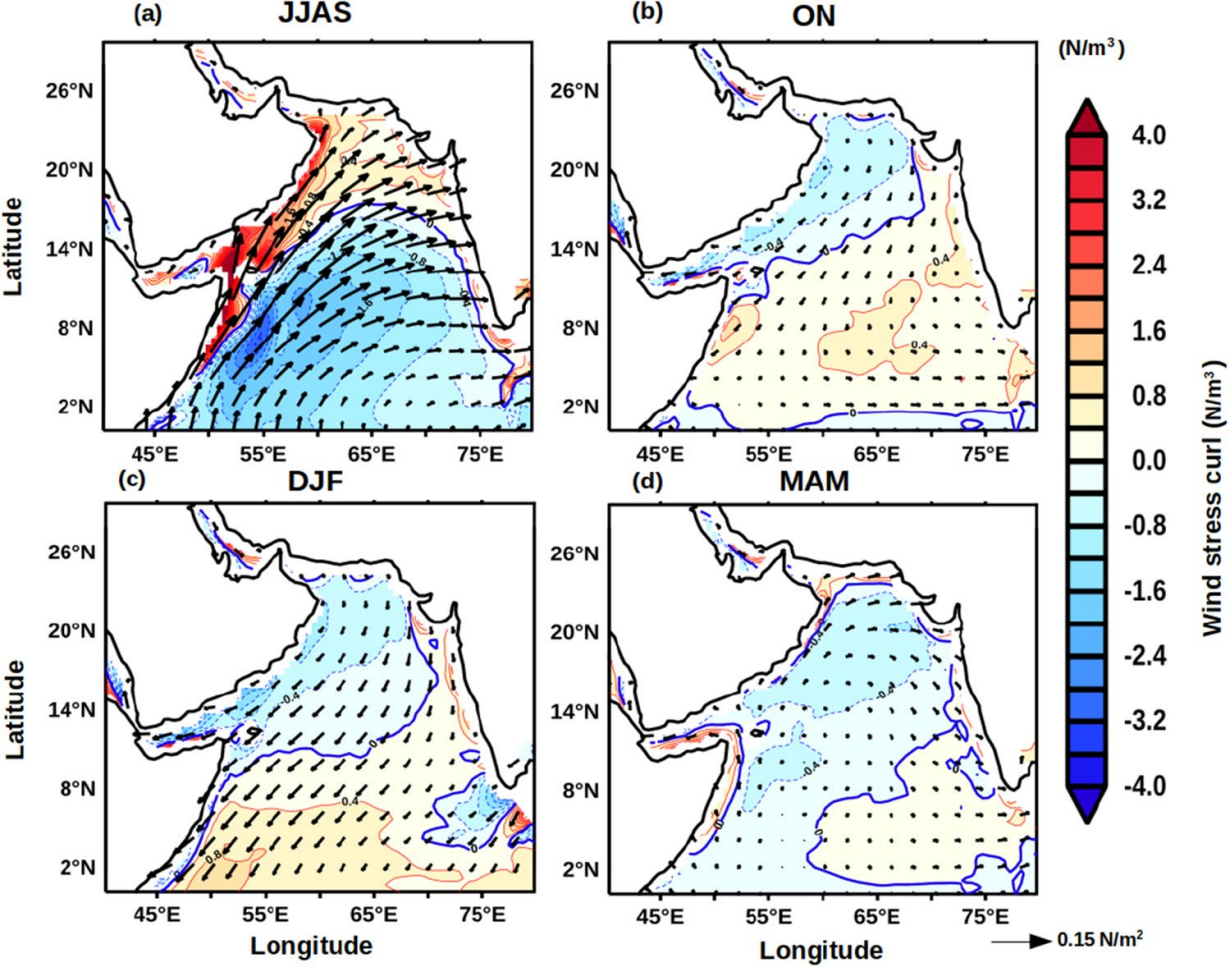
Annual cycle of climatology of wind stress curl (WSC, N/m^3^) during (**a**) JJAS, (**b**) ON, (**c**) DJF, and (**d**) MAM. Contour lines represent the magnitude of zero wind stress curl while the arrows represent the wind stress (N/m^2^) vector.

**Figure 4:**
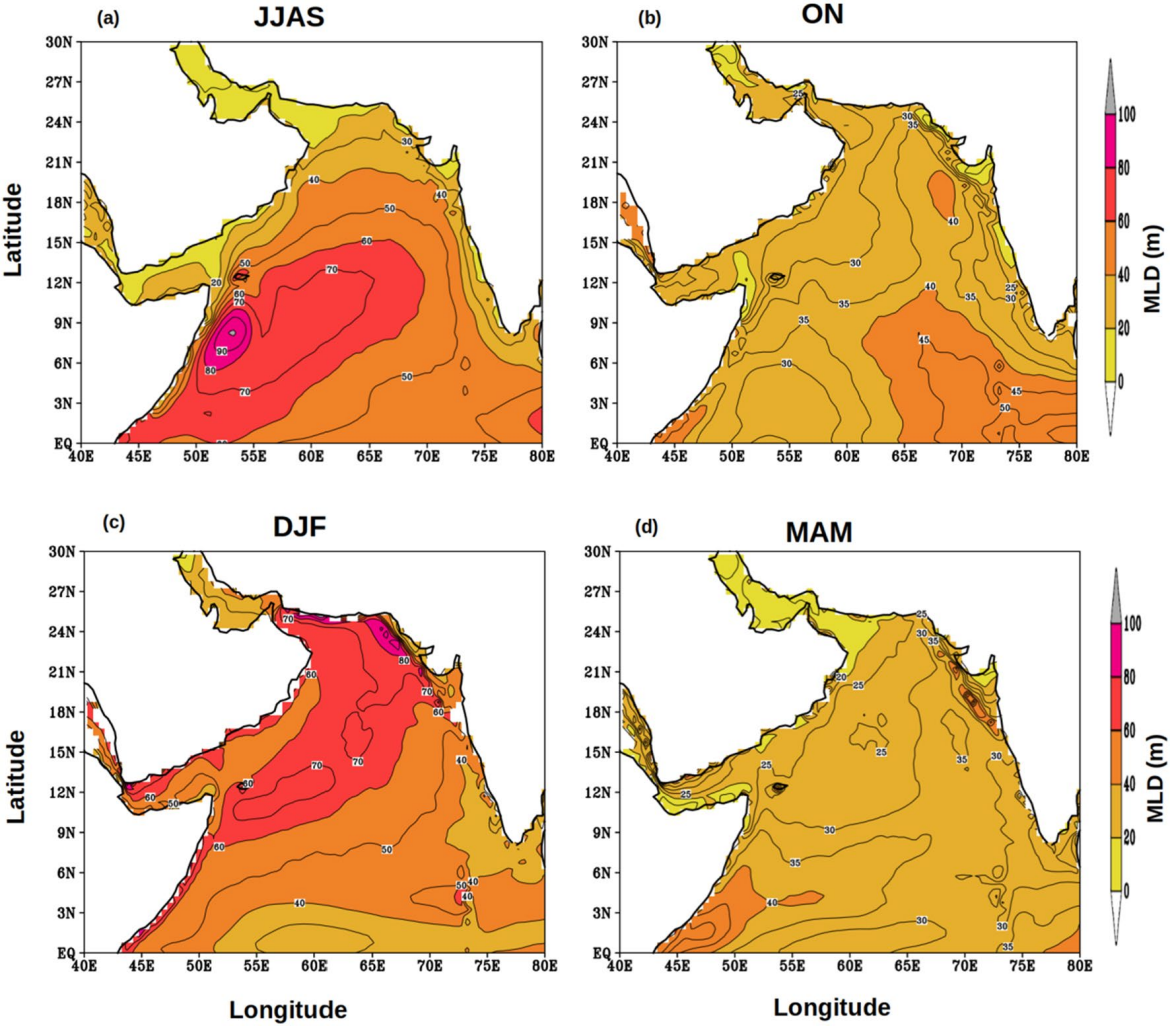
Annual cycle of climatology of mixed layer depth (MLD, m) during (**a**) JJAS, (**b**) ON, (**c**) DJF, and (**d**) MAM.

To decipher the impact of FJ on the upper ocean dynamics, the annual cycle of wind stress curl (WSC) (Fig. 3) and mixed layer depth (MLD) (Fig. 4) were examined in the AS. The axis of the FJ is denoted by the zero WSC in Fig. 3a during JJAS. North of the axis of the FJ the WSC was positive which would drive Ekman suction and support upwelling of subsurface waters. In contrast, south of the axis of the FJ the negative WSC would drive Ekman pumping and support downwelling of surface waters. The response of the wind forcing was seen clearly in the spatial distribution of MLD during JJAS (Fig. 4a) which showed shallow mixed layer north of the axis of FJ and deep mixed layer south of it. These results are consistent with earlier studies based on shipboard observation^20,21^ as well as mooring^23^. As the season changes from ISM to post-monsoon (ON), the FJ collapses and disappears with weak WSC values over the entire AS (Fig. 3b). Accordingly, the MLD also showed a decrease of 20–30 m (Fig. 4b). In the winter season (DJF), under the prevailing easterly trade winds the WSC over the north-western AS was weak and negative, while in the eastern and most of the southern AS the WSC was weak and positive (Fig. 3c) suggesting a weak downwelling and upwelling respectively. Consistent with the wind forcing the basin-wide MLD in the AS was deep in the north and shallow in the south (Fig. 4c). However, the deepening of MLD by 30 m in the north in comparison to post-monsoon was not entirely driven by wind as the WSC, though negative, was weak. The winter cooling and convective mixing that prevails in this season in the northern AS contributed to the deepening of MLD^25^. The basin-wide WSC in the AS further weakened during the pre-monsoon (MAM) season (Fig. 3d) as winds were weak and variable. This resulted in the occurrence of basin-wide shallow MLD (Fig. 4d).

**Figure 5:**
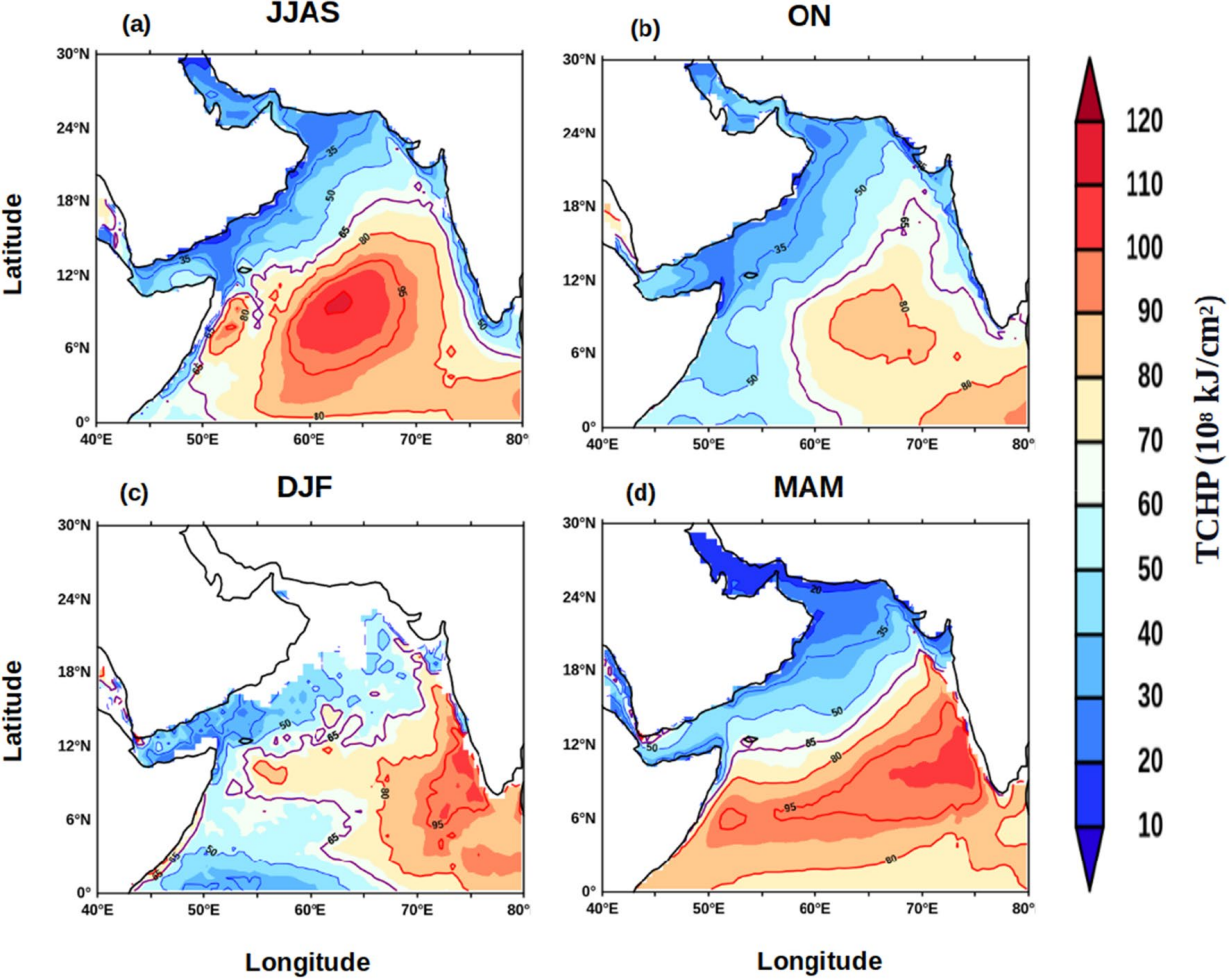
Annual cycle of climatology of UOHC (10^8 ^kJ/cm^2^) during (**a**) JJAS, (**b**) ON, (**c**) DJF and (**d**) MAM.

**Figure 6:**
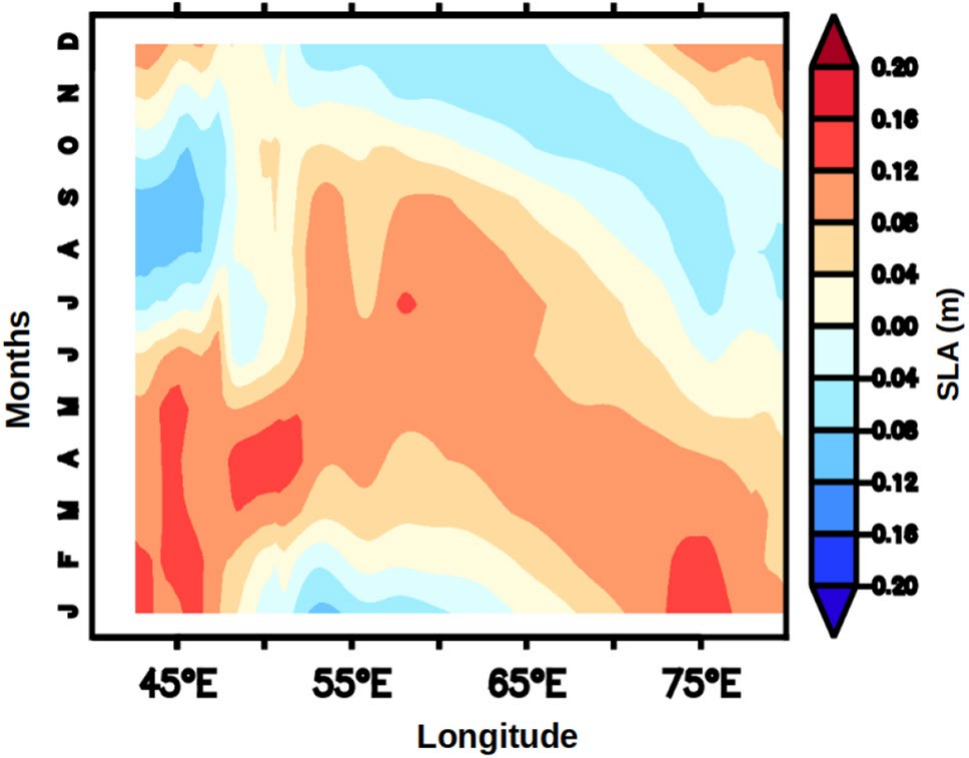
Hovmöller plot of climatology of monthly mean sea level anomaly (SLA, m) averaged over the latitudes from 4°N to 12°N in the Arabian Sea.

Thus, FJ plays a crucial role in basin-scale modulation of mixed layer during ISM. As the FJ modulated the mixed layer it is expected that the upper ocean heat content also will be impacted. The upper ocean heat content plays important role in the generation of several atmospheric processes including tropical cyclone^30^ and Indian summer monsoon^31^. The upper ocean thermal energy integrated from surface to the depth of 26 °C isotherm (D_26_) is a commonly used metric for the Upper Ocean Heat Content (UOHC) and generally referred as the tropical cyclone heat potential^32^. The seasonal cycle of UOHC (Fig. 5) was examined to understand the role of FJ in modulating the upper ocean heat content. The basin-wide structure of UOHC during ISM (JJAS) showed that south of the axis of FJ the value was more than 65 × 10^8^ kJ/cm^2^ and increased to 100 × 10^8^ kJ/cm^2^ towards the central AS, while towards the northwest and eastern parts of the AS it progressively decreased reaching as low as 20 × 10^8^ kJ/cm^2^ (Fig. 5a). The spatial distribution of D_26_ during ISM showed a similar pattern similar to that of UOHC (figure not shown) with values increasing form 65 m to 100 m south of the axis of the FJ, while towards the north and east it decreased and the lowest value was 20 m. Thus, the high values of UOHC were closely coupled to the deepening of D_26_. The spatial pattern of both UOHC and D26 was consistent with that of the WSC (Fig. 3). The region of high UOHC and D_26_ showed a progressive shift towards the southeast in the post-monsoon (Fig. 5b) and winter (Fig. 5c) monsoons. However, the magnitude of both UOHC and D_26_ showed a marginal decrease during post-monsoon, which was linked to the weakening of the WSC. In contrast, the magnitude of both UOHC and D_26_ increased during winter (Fig. 4d), though the WSC was positive. In the pre-monsoon season, both UOHC and D_26_ extend westward from their winter location and as a zonal band occupy the entire southern part of the AS. Interestingly, the WSC during this period was weak in the entire basin and positive over the southern and eastern parts. The increasing magnitudes of both D_26_ and UOHC during winter and its westward expansion until the pre-monsoon were an anomaly. A potential mechanism that could explain this anomaly is the propagation of Rossby waves. It is known that downwelling Rossby wave propagates from the west coast of India towards the coast of Somalia during each winter which is generated by the coastally trapped Kelvin wave traveling along the eastern boundary of the AS^22^. To explore this Hovmöller plot of SLA averaged over the latitudes 4° to 12°N were prepared (Fig. 6) which clearly indicated zonally sloping bands of SLAs. This was the signature of a westward propagating downwelling Rossby wave. These Rossby waves are known to deepen the thermocline and in the present case it was manifested by the deepening of the D_26_ and associated increase in the ocean heat content.

**Figure 7:**
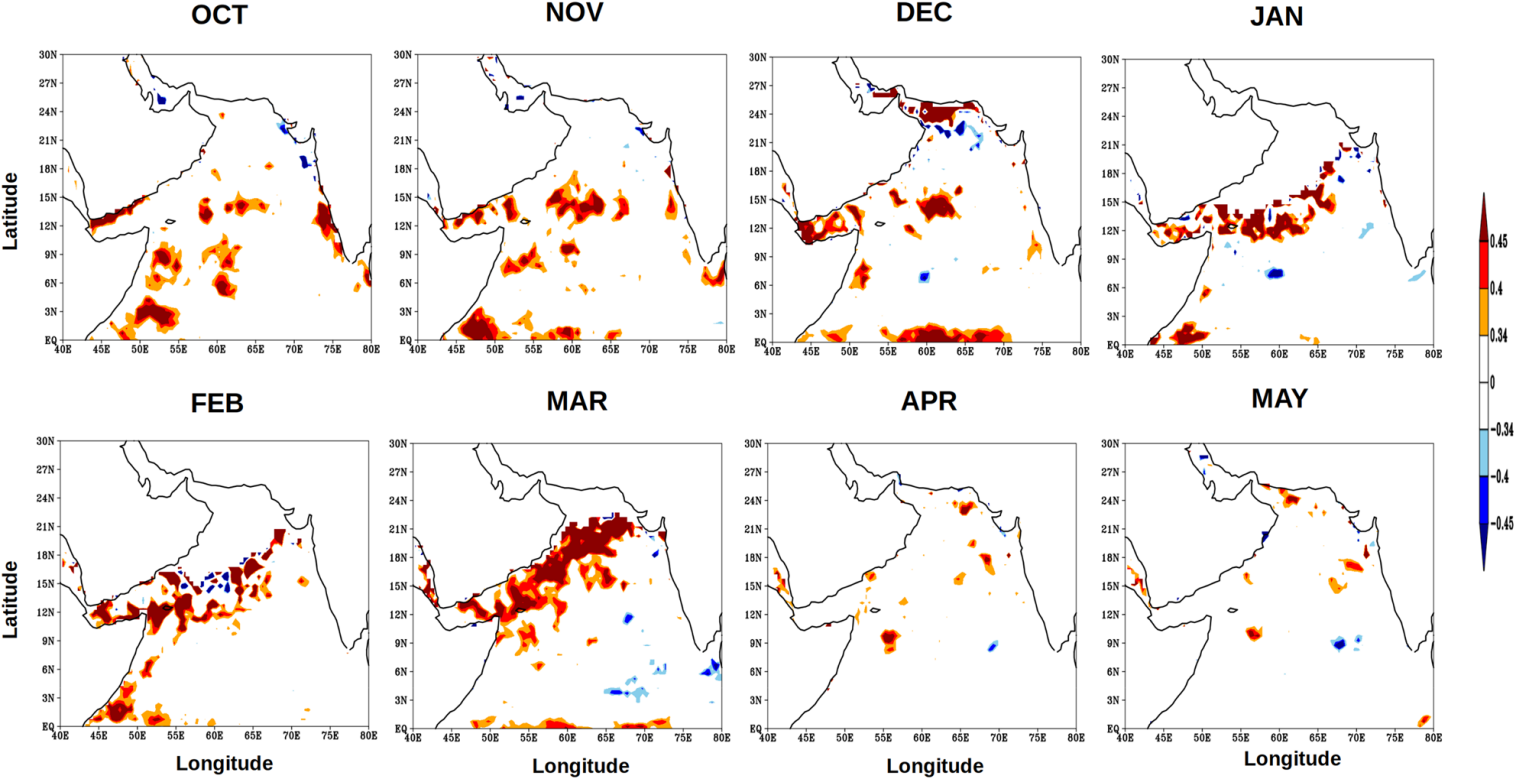
Monthly anomaly correlation of wind stress curl over the box region (50–70°E and 4–12°N, see Fig. 1) with UOHC of the Arabian Sea from October to May for the period of 1980–2015. The red colour represent the positive correlation value and the blue colour represents the negative correlation value, both significant at 95% confidence.

The above discussion leads to the understanding that due to the WSC associated with the FJ during ISM the southern part of the AS is able to store more thermal energy compared to the northern part of the AS. To further understand this association a box within the southern AS was selected (50–70°E and 4–12°N, Fig. 1), which essentially encompasses the region south of the axis of the FJ. A monthly spatial anomaly correlation was computed for the calendar months from October through May using the WSC within the box region with the UOHC in the AS during ISM to see how closely they were related. The monthly anomaly of WSC was calculated by subtracting the JJAS-averaged WSC within the box from each calendar month. Similarly the monthly anomaly of UOHC was calculated by subtracting the JJAS-averaged UOHC at each grid point from each calendar month. Figure 7 showed the monthly spatial anomaly correlation from October to May in the AS. The UOHC anomaly from October to December south of the FJ was positively correlated to the WSC at 95% confidence level. By the beginning of the subsequent year, i.e., during January and February, the region of significant positive correlations moved closer to the FJ axis. From March to May, the WSC and the UOHC anomaly in the southern AS were not significantly correlated at 95% confidence level. The result reiterates the role of FJ-induced Ekman dynamics in regulating the upper ocean heat content in the region south of the axis of the FJ during the post-monsoon and the winter monsoon.

**Figure 8:**
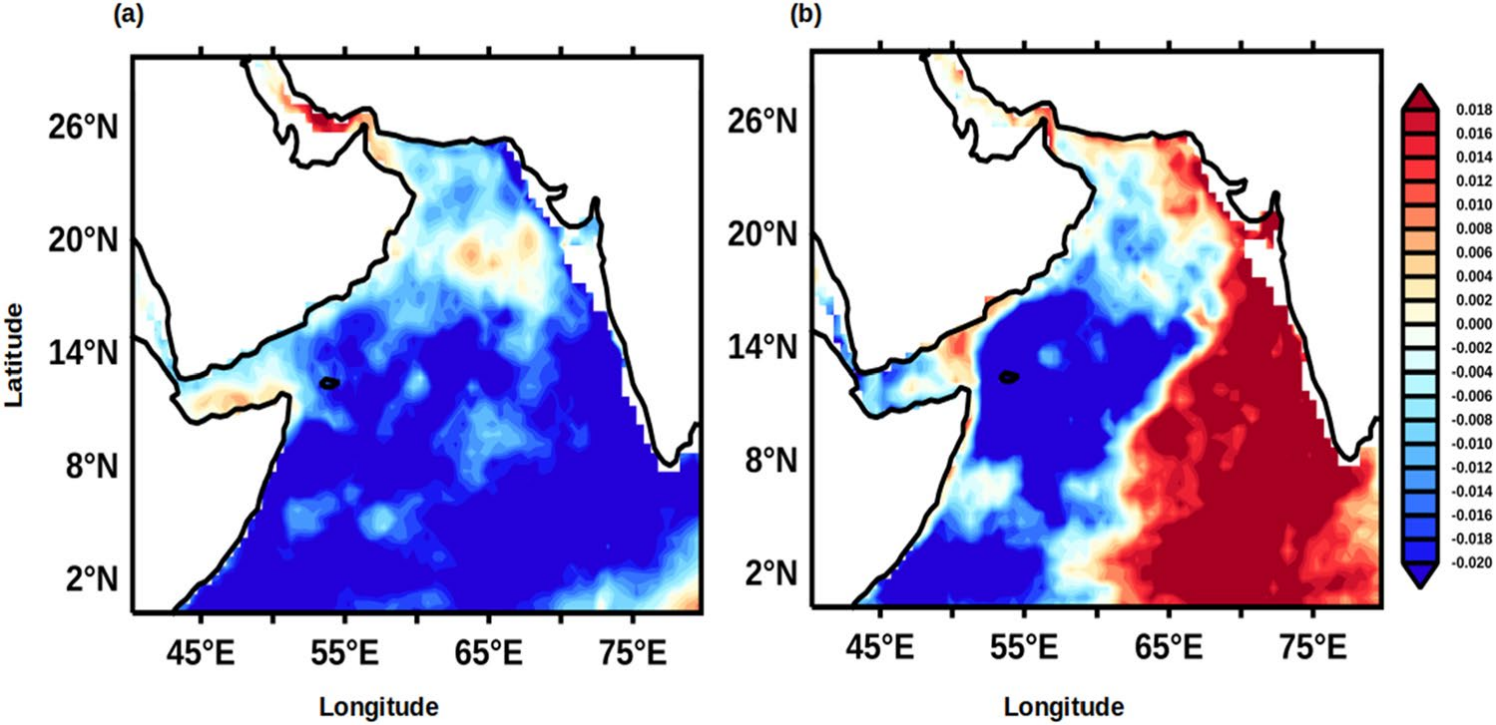
(**a**) The Principal Component 1 and (**b**) Principal component 2 of the EOF of the UOHC during ON.

To ensure the robustness of the above results, we have computed the variability of Empirical Orthogonal Function (EOF) for UOHC during ON months. Figure 8a,b shows the first and second principal components (PC) of the EOF. The variance of the first and second PCs of EOF for UOHC during ON months is 23.6% and 9.3% respectively. The second mode of EOF clearly brings out the pattern of spatial variability in the UOHC which is similar to that of the basin-wide pattern of WSC induced by the Findlater jet during ISM months. The contrast between the northern and the southern sides of the FJ axis is quite distinctly visible in the second PC of EOF which is shown in Fig. 8b.

**Figure 9:**
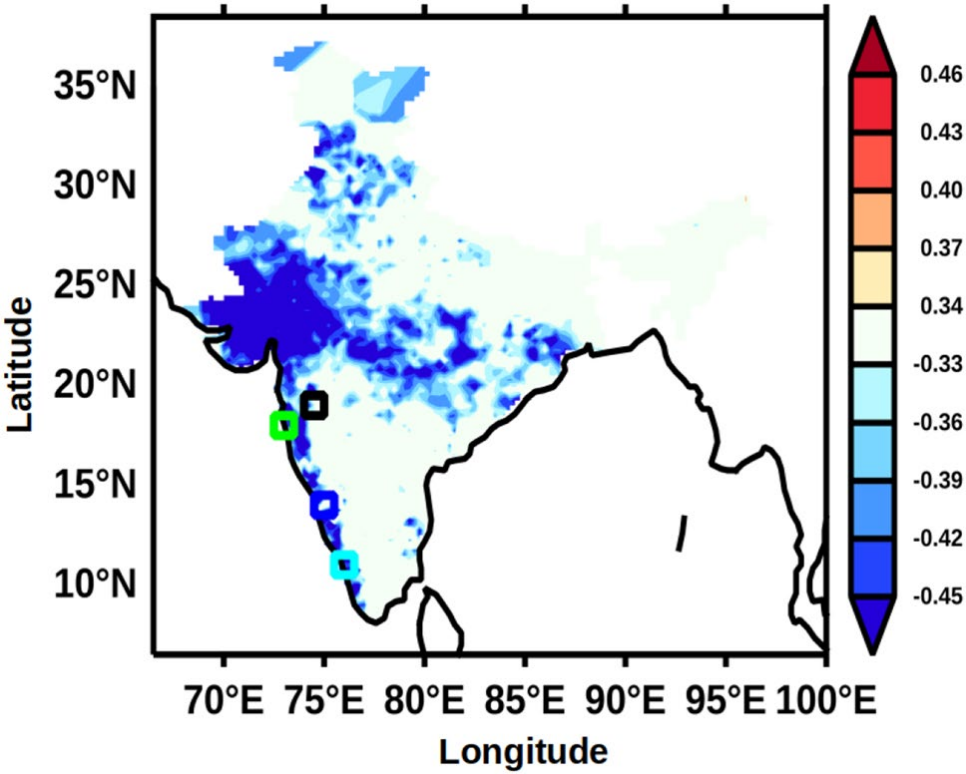
Anomaly correlation of WSC averaged during JJAS within the box region (50–70°E and 4–12°N, see Fig. 1) with rainfall averaged for the same months in the Indian sub-continent. The blue colour represents the significant negative correlation value at 95% confidence. The small squares of different colours represent the location of sub-divisonal stations, Madhya Maharashtra (black), Konkan (green), Coastal Karnataka (blue), and Kerala (cyan).

Having examined the relationship between the WSC within the box in the southern AS with UOHC over the AS, it is pertinent to see how the WSC within the box was related to the rainfall over the Indian sub-continent. Figure 9 presents the spatial correlation of WSC averaged within the box during June to September with rainfall averaged for the same period in the Indian sub-continent. The salient result is that the WSC over the box region is negatively correlated with rainfall along the western, central and northern parts of India at 95% confidence level. This implies that a higher negative WSC would lead to an increase in the ISM rainfall. Mechanistically, this happens through increase in the UOHC via deepened D_26_.

**Figure 10:**
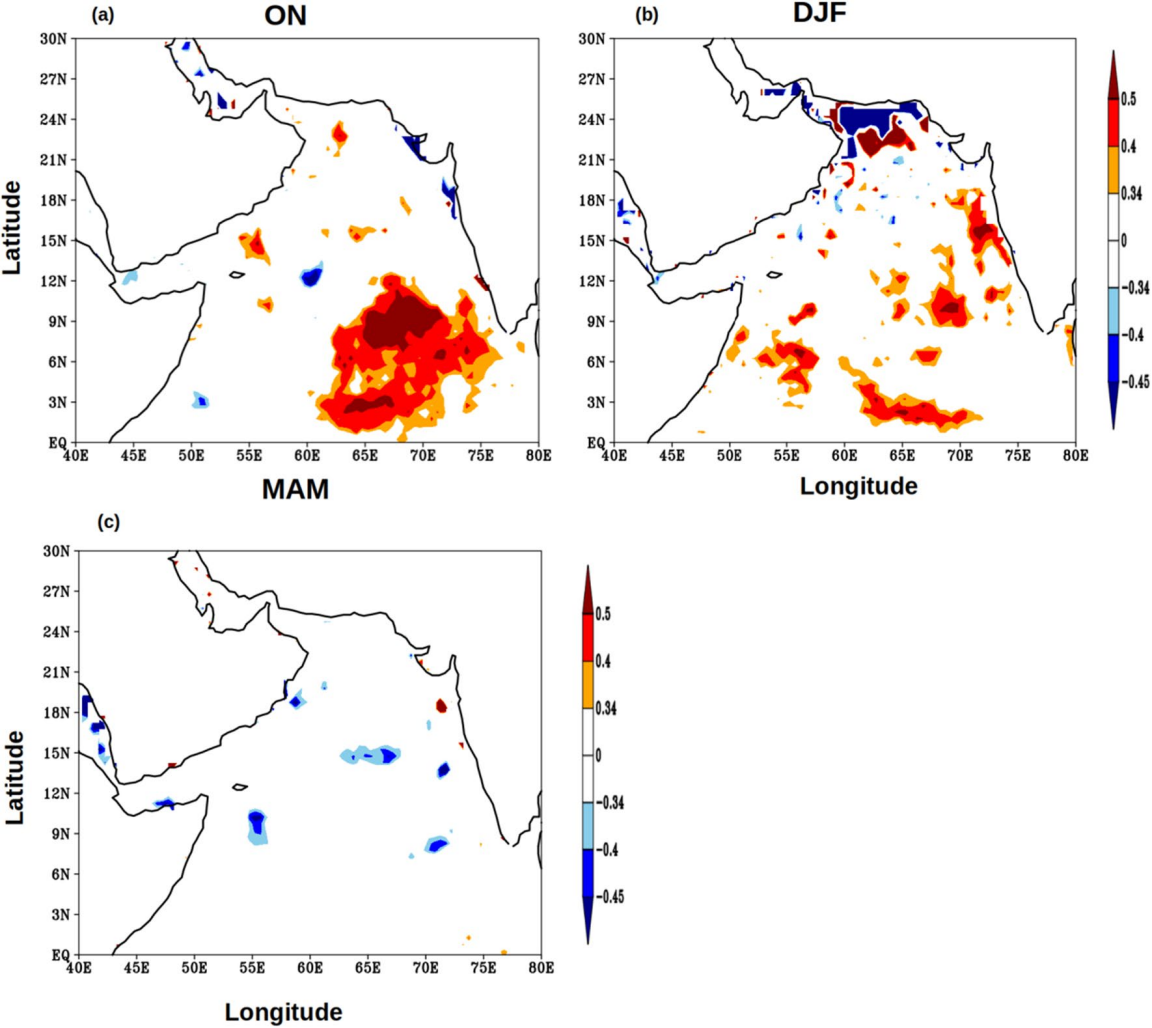
Monthly lead anomaly correlation of West Indian coastal rainfall (WICR) with UOHC in the Arabian Sea during (**a**) ON, (**b**) DJF, and (**c**) MAM for the period of 1980–2015. The red and blue colours represent the significant positive and negative correlation value respectively at 95% confidence.

Finally, to address how the upper ocean heat content was related to the Indian summer monsoon, the monthly mean climatology of rainfall data from four locations along the west coast of India (see Fig. 9 for locations) were averaged for June to September and designated as west Indian coastal rainfall (WICR). A lead correlation of WICR with UOHC in the AS were computed and presented in Fig. 10. In the post-monsoon period (Fig. 10a) the UOHC in the south-eastern part of the AS was positively and strongly correlated with WICR at 95% confidence level. During the winter monsoon period (Fig. 10b) the positive correlation of UOHC and WICR is spread over most part of the southern AS and along the west coast of India. The positive correlation of WICR and UOHC completely disappears in pre-monsoon period (Fig. 10c). The result from this lead correlation suggested that the UOHC in the southern part of the AS was anomalously higher during the subsequent seasons if the FJ strengthens and the rainfall over the WICR is more and vice versa. It emerges from the above results that the FJ signal was potentially ‘memorized’ in the sub-surface AS in the form of UOHC. It is intriguing why the heat-put by the summer monsoon into the subsurface layer of the southern AS was not reduced or dissipated by mixing in the subsequent two seasons. In the next paragraphs, based on the results from the present study and existing understanding of the regional oceanography of the AS, we propose a potential mechanism, which facilitates the observed ‘memory’ of the FJ to be preserved through the next two seasons.

During the ISM period the FJ produces a strong upper ocean convergence through the wind stress curl towards the south of its axis. The resultant Ekman pumping leads to the downwelling of water to the south of the FJ axis. The downwelling of warm surface waters during June to September eventually builds up the heat content in the upper thermocline. A stronger FJ would lead to an increased Ekman pumping and deeper downwelling. This in turn will deepen the D_26_ and results in the increase the upper ocean heat content during ISM. As the season changes from ISM to post-monsoon two processes co-occur in the AS: (1) the secondary heating and development of strong thermal stratification of the upper ocean, (2) the prevalence of weak and variable winds^1^. Both of these will curtail the mechanical mixing and results in retaining most of the heat in the subsurface layers in the upper thermocline.

Why does not the subsurface heat stored during post-monsoon come up in the winter under winter convection? In winter, surface waters of the northern AS experiences sensible heat loss by reduced incoming solar radiation and latent heat loss due to evaporative cooling under the prevailing dry north-easterly winds of continental origin both of which will deepens the mixed layer^24^. In contrast, in the southern part of the AS, especially south of 15°N do not experience winter cooling and convection. As the convective mixing is absent the heat stored in the subsurface does not dissipate. In addition, the westward propagating downwelling Rossby waves radiated from the eastern boundary of the AS deepens the upper thermocline. This will help to retain the warm waters below the surface during winter as has been inferred from the UOHC and deep D_26_ during winter. As the inherent strength of the winter monsoon winds over the AS is substantially weaker compared to the ISM, the wind-driven entrainment has very little effect on the subsurface warmer waters. All this keeps the FJ-induced heat at a deeper level during the post-monsoon and winter monsoon. Once the propagation of Rossby wave from the eastern AS collapses towards the end of winter monsoon/beginning of the pre-monsoon the thermocline shoals and the heat that was stored in the previous seasons in the subsurface layer becomes available to the upper ocean for subsequent ocean–atmosphere interaction.

## Summary and concluding remarks

The seasonal cycle of the AS is a net response to the Ekman dynamics associated with the local southwest and succeeding northeast monsoons, associated air-sea fluxes, and annual Rossby waves. Through an examination of various reanalysis/observational datasets for the period 1980–2015, we document a contiguous chain of inherent seasonal processes in the AS from boreal summer through the following year spring, which showed storage of the signature of the FJ in the form of heat energy in the sub-surface, represented by the UOHC for three consecutive seasons. Our study ascertains that the UOHC in the southern part of the AS during the winter monsoon was positively correlated with the WSC of the summer monsoon. Further, during a strong monsoon with strong FJ the UOHC in the southern AS was not only anomalously high, as expected, but continues to be so even during the subsequent boreal winter season in such years.

This THCP signature of the previous year FJ stored in the AS is because of the seasonality of winds over the AS and the westward propagating Rossby wave radiated from the eastern boundary of AS. The modulation of the upper thermocline by the Rossby wave keeps the signal stored in the subsurface till the beginning of boreal spring and probably may pre-condition the local SST for the next monsoon. Exploring this aspect is beyond the scope of the present study. The proposed mechanism of subsurface storage of summer monsoon memory in our study is based only on the correlation analysis and needs further extensive modelling studies over the AS to further understand the interactions of FJ with the subsurface ocean. Nevertheless, findings from this study have great implications on the understanding of dynamics and thermodynamics of the upper ocean in the AS in the context of summer monsoon and its predictability.

## Data and methodology

The zonal and meridional component of wind at different pressure levels having a horizontal resolution of 25 km was downloaded for the period 1980 to 2015 from the National Centre for Medium Range Weather Forecast (NCMWRF) monthly reanalysis data product^33^ (https://rds.ncmrwf.gov.in/dashboard/download). This data was used to calculate the climatological monthly mean wind at different pressure levels to characterize the FJ in the AS.

The subsurface temperature (^o^C) and wind stress ((N/m^2^) data were taken from the Simple Ocean Data Assimilation Ocean/sea ice reanalysis (SODA) Version 3.3.1^34^ for the period 1980 to 2015 which has a spatial resolution of 0.25° × 0.25° longitude by latitude (https://www2.atmos.umd.edu/~ocean/index_files/soda3.3.1_mn_download.htm). The climatological monthly mean wind stress curl was calculated for the box 50°E to 70°E and 4°N to12°N in the central AS (see Fig. 1) following the equation^35^

1 $$WSC=\frac{\partial {\tau }_{y}}{\partial x}-\frac{\partial {\tau }_{x}}{\partial y}$$

where ${\tau }_{x}$ and ${\tau }_{y}$ respectively denote the wind stress components along zonal and meridional directions.

The subsurface temperature data was used for the determination of the monthly mean climatology of depth of the 26 °C isotherm (D26) and further for the calculation of the monthly mean climatology of tropical cyclone heat potential referred as Upper Ocean Heat Content (UOHC)^32^ using the following equation

2 $$UOHC=\rho *Cp{\int }_{0}^{D26}\left[T\left(z\right)-26\right]dz$$

where T(z) is the temperature at depth z (m), ρ is the density (1024 kg/m^3^), and C_p_ is the specific heat (3850 J/kg C) of sea water.

The sea level anomaly (SLA, m) data having a spatial resolution of 0.25° × 0.25° longitude by latitude for the period 1993–2019 was downloaded from the Copernicus Marine Environment Monitoring Services^36^ (https://resources.marine.copernicus.eu/). Using this data the monthly mean climatology of SLA was computed for the preparation of Hovmöller plot.

The gridded rainfall data over India having a spatial resolution of 0.25° × 0.25° longitude by latitude for the period 1980–2015 was obtained from India Meteorological Department (IMD)^37^ (https://cccr.tropmet.res.in/home/data_portals.jsp). This data was used for calculating the climatology of average Indian summer monsoon rainfall during June to August (JJAS) at each of the grid point. Additionally, we have used the rainfall data from four sub-divisional stations from Madhya Maharashtra, Kerala, Konkan and Goa, and Coastal Karnataka (see Fig. 1 for location) for the same period. The rainfall data of the four sub-divisional stations were averaged for June to August (JJAS) for the period 1980–2015 and referred as West Indian coastal rainfall (WICR) for the present study.

We have de-trended the data for the study period to remove any linear trends present in the data. Furthermore, we used the linear anomaly correlation analysis and the statistical significance of the correlations was obtained through a 2-tailed Student’s t-test.

## Data Availability

All the data used in the study were downloaded from the open source and the web site details are given under “Data and methodology”.
